# Tradeoffs between proliferation and transmission in virus evolution– insights from evolutionary and functional analyses of SARS-CoV-2

**DOI:** 10.1186/s12985-025-02727-5

**Published:** 2025-04-19

**Authors:** Jui-Hung Tai, Ding-Chin Lee, Hsin-Fu Lin, Tai-Ling Chao, Yongsen Ruan, Ya-Wen Cheng, Yu-Chi Chou, You-Yu Lin, Sui-Yuan Chang, Pei-Jer Chen, Shiou-Hwei Yeh, Hurng-Yi Wang

**Affiliations:** 1 Graduate Institute of Clinical Medicine, College of Medicine, National Taiwan University, Taipei, 10002 Taiwan; 2 Genome and Systems Biology Degree Program, National Taiwan University and Academia Sinica, Taipei, 10617 Taiwan; 3 Department of Microbiology, College of Medicine, National Taiwan University, Taipei, 10617 Taiwan; 4 Department of Clinical Laboratory Sciences and Medical Biotechnology, College of Medicine, National Taiwan University, Taipei, 10002 Taiwan; 5 State Key Laboratory of Biocontrol, School of Life Sciences, Southern Marine Science and Engineering Guangdong Laboratory (Zhuhai), Sun Yat-sen University, Guangzhou, China; 6 Biomedical Translation Research Center (BioTReC), Academia Sinica, Taipei, 11529 Taiwan; 7 Hepatitis Research Center, National Taiwan University College of Medicine and National Taiwan University Hospital, Taipei, 10002 Taiwan; 8 Department of Internal Medicine, National Taiwan University College of Medicine, National Taiwan University Hospital, Taipei, 10002 Taiwan; 9 Department of Medical Research, National Taiwan University College of Medicine, National Taiwan University Hospital, Taipei, 10002 Taiwan; 10 Institute of Ecology and Evolutionary Biology, National Taiwan University, Taipei, 10617 Taiwan; 11 Graduate Institute of Medical Genomics and Proteomics, National Taiwan University College of Medicine, Taipei, 10002 Taiwan

**Keywords:** Pleiotropy, Genetic diversity, Spike M1237I, Frequency spectrum, Epistatic interactions

## Abstract

To be successful, a virus must maintain high between-host transmissibility while also effectively adapting within hosts. The impact of these potentially conflicting demands on viral genetic diversity and adaptation remains largely unexplored. These modes of adaptation can induce uncorrelated selection, bring mutations that enhance certain fitness aspects at the expense of others to high freqency, and contribute to the maintenance of genetic variation. The vast wealth of SARS-CoV-2 genetic data gathered from within and across hosts offers an unparalleled opportunity to test the above hypothesis. By analyzing a large set of SARS-CoV-2 sequences (~ 2 million) collected from early 2020 to mid-2021, we found that high frequency mutations within hosts are sometimes detrimental during between-host transmission. This highlights potential inverse selection pressures within- versus between-hosts. We also identified a group of nonsynonymous changes likely maintained by pleiotropy, as their frequencies are significantly higher than neutral expectation, yet they have never experienced clonal expansion. Analyzing one such mutation, spike M1237I, reveals that spike I1237 boosts viral assembly but reduces in vitro transmission, highlighting its pleiotropic effect. Though they make up about 2% of total changes, these types of variants represent 37% of SARS-CoV-2 genetic diversity. These mutations are notably prevalent in the Omicron variant from late 2021, hinting that pleiotropy may promote positive epistasis and new successful variants. Estimates of viral population dynamics, such as population sizes and transmission bottlenecks, assume neutrality of within-host variation. Our demonstration that these changes may affect fitness calls into question the robustness of these estimates.

**Supplementary Information:**

The online version contains supplementary material available at 10.1186/s12985-025-02727-5.

## Background

Throughout its lifecycle, a virus encounters two primary challenges. On one hand, it must ensure high transmissibility to efficiently spread among hosts. On the other hand, it must maintain a high replicative capability and adapt to the local environment within a host during infection [1, 2]. Mutations that increase viral replication rates might affect transmission or vice versa, as selection pressures during transmission and throughout the infection can differ markedly [3, 4]. Some variants might excel at transmission and colonization, whereas others excel at escaping host immune selection. However, it is rare for single mutations to simultaneously improve transmission, colonization, and host immune system escape [2]. For example, it has been shown that escape mutations in HIV driven by CTL pressure can revert to wild-type after transmission to individuals without the selecting HLA alleles [4, 5].

Hou et al. tracked the evolution of SARS-CoV-2 in a cohort of 79 COVID-19 patients with complete contact records and found that advantageous new mutations emerge regularly within individual hosts but rarely succeed in spreading among hosts [6]. This finding hints at a possible disconnect, or even antagonism, between selection pressures inside a host versus those during transmission between hosts, potentially affecting long-term virus evolution. For instance, variants maintained by antagonistic selection incur fitness costs and/or may facilitate epistatic interactions and promote virus adaptation [7]. Moreover, parameters of viral dynamics models are estimated based on the assumption that observed variants are selectively neutral [8, 9]. If a significant portion of mutations affects fitness, the accuracy of these estimates would be compromised. Although some studies have examined these dynamics in viruses causing chronic infections, such as HIV [10–12], the impact of potentially conflicting pressures - arising from the dual demands of the viral life cycle - on genetic diversity and adaptation remains largely unexplored in viruses that cause acute infections. This gap in understanding is partly due to a scarcity of suitable data.

The overwhelming number of SARS-CoV-2 sequences provides an unprecedented opportunity to track evolution in ways unimaginable in the study of any other living organisms. To investigate the role of potential pleiotropic effects induced by the dual demands of the viral life cycle, we analyzed a large set of SARS-CoV-2 genome sequences (~ 2 million) collected between 2020 and 2021. Our observations indicate that mutations prevalent within hosts often face challenges during inter-host transmission, highlighting evolutionary conflict within- and between-hosts. We also identified a set of nonsynonymous changes likely sustained by inverse pleiotropy. Their frequencies surpass those at four-fold degenerate sites, where changing the third base of a codon to any of the four nucleotides does not alter the encoded amino acid. Despite their relatively high frequency, none of these changes have undergone clonal expansion. Analyzing one such mutation, spike M1237I, reveals that spike I1237 boosts viral assembly but reduces in vitro transmission, highlighting its antagonistic effect. Although they make up about 2% of total changes, these variants represent 37% of genetic diversity. Further analysis shows that they are significantly enriched in novel Omicron strains, suggesting that they might interact with each other to reduce antagonistic effect [7], or even compensate for one another’s deleterious effects through positive epistasis. Consequently, pleiotropic interaction may play an important role in shaping viral genetic variation and adaptation.

## Methods

### SARS-CoV-2 genome collection and analysis

The data collection and preprocessing are as previously described [13]. In brief, we downloaded 1,929,395 SARS-CoV-2 genomes from the GISAID database (https://www.gisaid.org/) as of July 5, 2021 and aligned to the Wuhan-Hu-1 reference sequence (EPI_ISL_402125) using MAFFT [14] (--auto --keeplength). We used snp-sites (-v; [15]) to identify single nucleotide polymorphisms (SNPs) and *bcftools* (merge -force-samples -O v) to merge the vcf files. We identified 65,673 SNPs in coding regions. Because our dataset includes most of the possible mutations at each site, mutation counts in each category mainly reflect the nucleotide composition of the virus genome and do not directly reflect mutation prevalence, thus the frequency of nucleotide change was used as a proxy to estimate the mutation prevalence across types.

To define high frequency mutations, we first calculated average mutation frequency of four-fold degenerate sites (7.4 × 10^− 4^). With 4,236 four-fold degenerate sites and standard deviation of 1.26 × 10^− 2^, the 95% confidence interval for four-fold degenerate site frequency is 3.6 × 10^− 4^ to 1.1 × 10^− 3^. Mutations that appeared more often than 10^− 3^ are considered as high frequency for convenience.

For intra-host variation of SARS-CoV-2, publicly available high-throughput sequencing data sets were downloaded from the NCBI Sequence Read Archive to assess intra-host genetic variation (before July 2020). Data set IDs are listed in Table S1. Bioinformatics processing was basically following Lythgoe, Hall [16]. In brief, all sequence reads pairs were classified by Kraken version2 using Human, Bacteria and Viral database (pulled Feb 2024). Sequences identified as viral and those unclassified reads were kept by custom python script. Sequence reads were then removed Illumina adapter sequences using Trimmomatic version 0.39, with the ILLUMINACLIP options set to “2:10:7:1:true MINLEN:80”. Trimmed reads were mapped to the SARS-CoV-2 reference genome Wuhan-Hu-1 (EPI_ISL_402125) using smalt with default options. The *bcftools* [17] was used for intra-host variation calling. To mitigate sequencing errors, only reads with mapping quality ≥ 20 and sequence depths ≥ 100 were considered. The fixed polymorphisms (frequency ≥ 0.95) were discarded and only consider SNP frequency ≥ 0.03 as intra-host variation. All further calculations were performed using R and in-house Python scripts.

For mutations of Omicron strain BA.2, we compared USA-WA-S16116 (EPI_ISL_8822434) with the reference genome and retrieved 46 nonsynonymous mutations for further analysis.

### Calculate Watterson estimator of genetic diversity

The Watterson estimator of genetic diversity, θ [18], was calculated by randomly selecting 50, 100, and 1,000 SARS-CoV-2 genomes from the dataset. To demonstrate the influence of mutations of different frequencies on viral genetic diversity, we individually removed mutations falling into a set of frequency bins and calculated θ. The process was repeated 100 times and the mean and standard deviation of θ were estimated. The results for selecting 50, 100, and 1,000 SARS-CoV-2 genomes are similar. Therefore, we only present the results based on selecting 100 sequences.

### SARS-CoV-2 viruses

Thirty-eight virus isolates were obtained from the sputum of SARS-CoV-2-infected patients, propagated in Vero E6 cells in Dulbecco’s modified Eagle’s medium (DMEM) supplemented with 2 µg/mL tosylsulfonyl phenylalanyl chloromethyl ketone-trypsin. Virus isolates used in the current study have been deposited in the GISAID platform and accession numbers are listed in Table S2.

### Plaque forming assay

The plaque assay was performed as described previously [19]. In brief, Vero E6 cells (2 × 10^5^ cells/well) were seeded in triplicate in 24-well tissue culture dishes in DMEM supplemented with 10% fetal bovine serum (FBS) and antibiotics. After 24 h post-infection, virus-containing culture supernatant was added to the cell monolayer for 1 h at 37 °C, which was then washed with PBS and maintained with 1% methylcellulose medium. After incubation for 5 days, cells were fixed with 10% formaldehyde overnight and stained with 0.7% crystal violet for plaque counting. Plaque forming activity was estimated from three independent experiments.

### Plasmid constructs

The humanized SARS-CoV-2 spike expression plasmid in the pcDNA3.0-HA vector, SCoV2-S, and the mutated SCoV2-S-D614G was constructed as described previously [20]. To construct the expression plasmids for the spike of Alpha variant (B.1.1.7), pcDNA3.1(+)-SCoV2-S(B.1.1.7), we purchased the synthetic DNA fragment encoding the spike of Alpha variant virus (B.1.1.7) from Integrated DNA Technologies (IDT) for cloning into the KpnI and EcoRI restriction enzyme sites of pcDNA3.1(+) expression vector (Thermo Fisher). The construct contains the representative mutations of the Alpha variant virus, with 69–70 del, Y144 del, N501Y, A570D, D614G, P681H, T716I, S982A, and D1118H. To optimize pseudotyped lentivirus production, pcDNA3.1(+)-SCoV2-S(B.1.1.7)-Δ18aa was constructed [21]. The mutant M1237I spike related constructs were generated using the QuikChange II site-directed mutagenesis kit (Agilent) with the primer set of S-M1237I-F: 5’-ACAGCAGGATGTTATACAGCACAGCATGATGGTCAC-3’ and S-M1237I-R: 5’-GTGACCATCATGCTGTGCTGTATAACATCCTGCTGT-3’. The plasmids expressing SARS-CoV-2 proteins, pLVX-EF1alpha-SARS-CoV-2-M (membrane) and pLVX-EF1alpha-SARS-CoV-2-E (envelope), with 2× streptavidin tag at the C-terminus were kindly provided by Dr. Chia-Wei Li from the Institute of Biomedical Sciences Center, Academia Sinica, Taiwan.

### Cell culture experiments

Human ACE2-expressing 293T stable cell line (293T-hACE2) were kindly provided by Dr. Mi-Hua Tao (Institute of Biomedical Sciences Center, Academia Sinica, Taiwan). The 293T, 293T-hACE2, and Calu-3 cells were maintained in DMEM (10–15% FBS) [22]. The plasmid was transfected into 293T cells with Lipofectamine™ 2000 (Thermo Fisher) and the cells were harvested 24 h post-transfection for subsequent analysis.

### Cell-cell fusion assay

A quantitative GAL4-based mammalian luciferase reporter assay was previously established to assess cell-cell fusion activity [20], containing a reporter construct (pGAL4/UAS-TK-Luc) and a transcriptional activator construct (pGAL4DBD-hAR-NTD). To detect cell-cell fusion activity mediated by WT-S and M1237I-S protein, 293T-hACE2 cells transfected with pGAL4/UAS-TK-Luc were prepared as target cells; 293T cells expressing the either WT-S or M1237I-S and pGAL4DBD-hAR-NTD were prepared as effector cells. After 24 h transfection, 293T cells expressing GAL4DBD-hAR-NTD protein and S protein were trypsinized and seeded on 293T-hACE2 cells expressing the GAL4/UAS-TK-Luc protein (293T: 293T-hACE2 ratio = 1:3). The cells were co-cultured for 20 h and harvested to measure the fusion activity by detecting the luciferase assay following the manufacturer’s instruction (Promega).

### Production and purification of SARS-CoV-2 Spike pseudotyped lentiviruses

Pseudotyped lentiviruses carrying various SARS-CoV-2 spike proteins were generated by transiently transfecting 293T cells with pCMV-DR8.91, pLAS2w.Fluc.Ppuro, and pcDNA3.1-SCoV2-S(B.1.1.7) related spike expression constructs using TransITR-LT1 transfection reagent (Mirus). Culture media were refreshed at 16 h and harvested at 48 h and 72 h post-transfection. Cell debris was removed by centrifugation at 4,000 x g for 10 min; the supernatant was passed through 0.45-mm syringe filter (Pall Corporation) and the pseudotyped lentiviruses were aliquoted and stored at -80 °C.

### Estimation of lentiviral titers using the alarmablue assay

The transduction unit (TU) of SARS-CoV-2 pseudotyped lentiviruses were estimated by using a cell viability assay in response to limiting lentivirus dilution. In brief, 293T-hACE2 cells (stably expressing human ACE2) were plated on 96-well plates one day before lentivirus transduction. To determine the titer of the pseudotyped lentivirus, different amounts of lentivirus were added to the culture medium containing polybrene (final concentration 8 mg/ml). Spin infection was carried out at 1,100 x g in 96-well plate for 30 min. After incubating at 37 °C for 16 h, culture media containing viruses and polybrene were replenished with complete DMEM containing 2.5 µg/ml puromycin. The culture media were discarded 48 h post-treatment and cell viability was estimated using 10% AlarmaBlue reagents according to manufacturer’s instructions (Thermo Fisher). Viral titers (transduction units) were determined by plotting cell survival against diluted viral dose, with uninfected cells (without puromycin treatment) survival rate as 100%.

### SARS-CoV-2-virus like particle (SC2-VLP) production and infection

SC2-VLPs containing luciferase (Luc)-encoding transcripts enveloped with S-I1237 or S-M1237 were prepared as previously described [23], with minor modifications. In brief, the Luc-T20 expressing construct (Addgene) were co-transfected with plasmids expressing the SARS-CoV-2 nucleocapsid (nCoV-2-N), membrane and envelope (CoV2-M-IRES-E, Addgene), and spike (nCoV-2-B117 or B117/M1237I) into the packaging 293T cells (with molar ratio for Luc-T20: N: M/E: S as 3: 1: 1: 1) with Lipofectamine™ 2000 (Thermo Fisher). At 24- and 48-hours post-transfection, culture media were collected and filtered through 0.45 µm filters, followed by viral titer and infectivity determination. For viral titer determination, the supernatant was first treated with 6000 U micrococcal nuclease (NEB) before viral RNA extraction with MagNA Pure (Roche Diagnostics) and reverse transcribed using SuperScript III Reverse Transcriptase System (Thermo Fisher). Quantitative PCR was performed using FastStart DNA SYBR Green on LightCycler 1.5 (Roche Diagnostics), with primer set 5’-AGACAGTGGTTGCCTACGGG-3’ and 5’-ATGCGAAGTGTCCCATGAGC-3’. For infectivity determination, supernatants containing equal amounts of Luc-VLP (MOI = 0.05) were processed to infect 293T-hACE2 cells. 24 h later, the cells were lysed using a passive lysis buffer (Promega) and equal amounts of lysates were used for luciferase reporter assays following the manufacturer’s instructions (Promega).

### Western blot analysis

Western blotting was performed using sodium dodecyl sulfate polyacrylamide gel electrophoresis (SDS-PAGE) and Western Lightning Plus-ECL (PerkinElmer) as previously described [24]. Antibodies used are as follows: rabbit anti-SCoV/SARS-CoV-2 nucleocapsid (generated by our laboratory), mouse anti-SARS-CoV/SARS-CoV-2 spike [1A9] (Genetex, GTX632604), rabbit anti-SARS-CoV-2 membrane (Novus Biologicals, NBP3-05698), rabbit anti-SARS-CoV-2 envelope (Cell signaling, #74698), rabbit anti-GAPDH (Genetex, GTX100118), horseradish peroxidase-conjugated mouse IgG (Genetex, GTX213111-01), and rabbit IgG (Genetex, GTX213110-01). Quantitative comparisons of viral protein amounts on the blots were made using VisionWorks Life Science Image Analysis software (UVP, Upland, CA USA).

### Statistical analysis

The plaque forming units quantified by plaque assays in triplicate are shown as the mean ± SD. Results from the cell-cell fusion reporter assay are shown as data representative of three independent experiments and presented as mean ± SD. Differences in data from the virus titer and fusion reporter assay between each indicated paired groups were evaluated by Student’s *t*-test. A *P* value ≤ 0.05 was considered statistically significant (*, *P* < 0.05; **, *P* < 0.01; ***, *P* < 0.001).

## Results

### Absence of correlation between intra-host and population-wide nonsynonymous variants

**Fig. 1:**
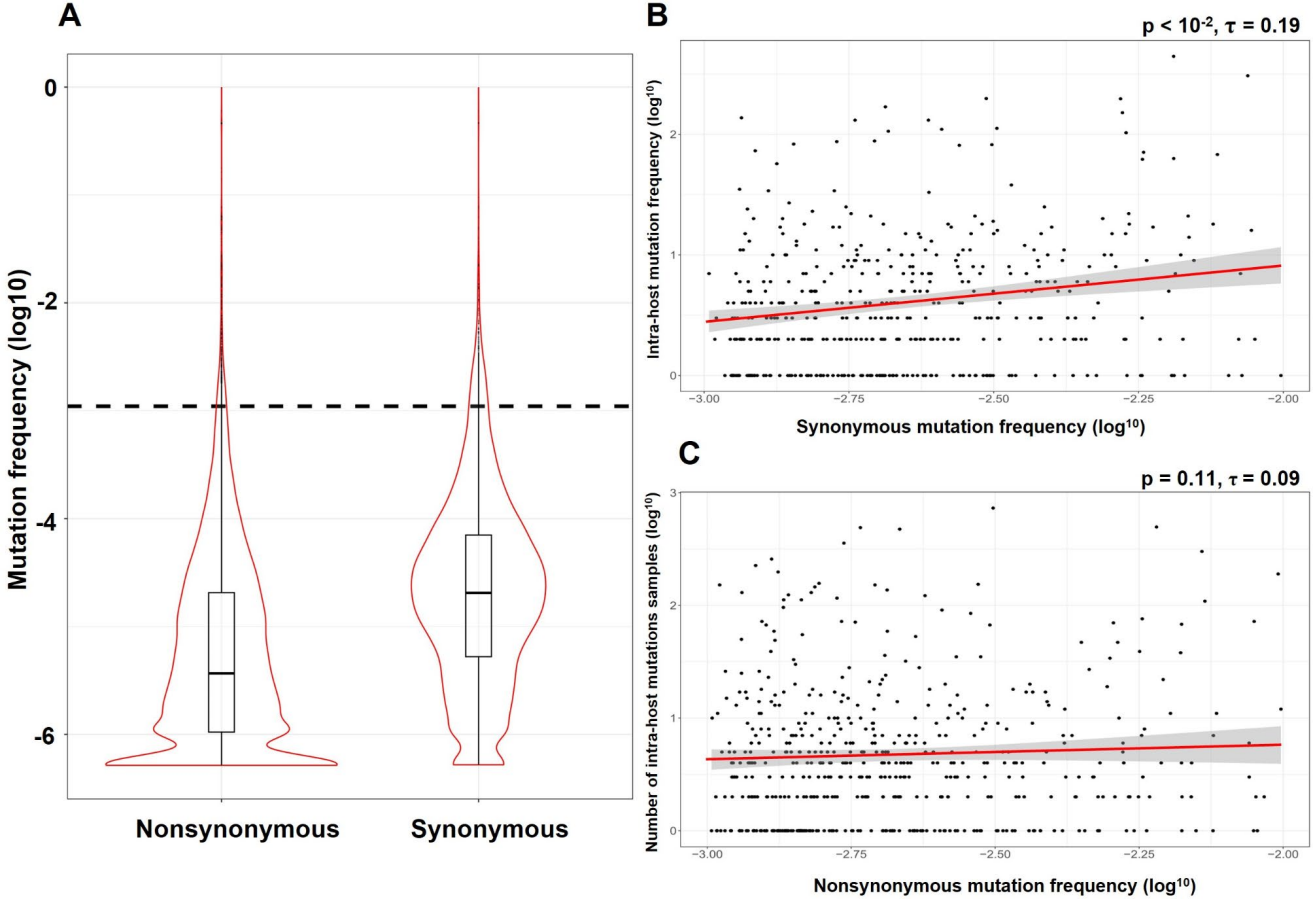
Frequency distribution of nonsynonymous (N) and synonymous (S) mutations in SARS-CoV-2. (**A**) Frequency distribution of synonymous and nonsynonymous mutations. Dashed line represents average frequency of four-fold degenerate sites. (**B**) Correlation of synonymous (*p* < 10^− 2^, Kendall correlation, τ = 0.19) and (**C**) nonsynonymous (lower panel, *p* = 0.11, τ = 0.09) mutation frequency between hosts (X-axis) with number of intra-host variants (Y-axis) (see Materials and Methods)

We identified 65,455 mutations from 28,363 sites out of 29,016 nucleotides in the coding regions of the SARS-CoV-2 genome. Most mutations are at low frequencies with an average of 4 × 10^− 4^ but a median value of 6.3 × 10^− 6^. The mean frequency is 4.6 × 10^− 4^ for 17,381 synonymous mutations and 3.8 × 10^− 4^ for 48,074 nonsynonymous (amino acid sequence altering) mutations (Fig. 1A).

To investigate whether virus evolution patterns are similar within- and between-hosts, we first accessed the intra-host genomic diversity of SARS-CoV-2 (Materials and Methods). Since mutations with frequencies exceeding 10^−^², such as those observed in the Alpha and Delta variants, are typically subject to positive selection (see latter), and those with frequencies below 10^−^³ are restricted to a small number of individuals, this analysis focused only on mutations with frequencies ranging from 10^−^³ to 10^−^². Synonymous mutation frequencies are positively associated with intra-host variation (*p* < 10^− 2^) (Fig. 1B), suggesting that evolution within- and between-hosts is correlated. However, this association is absent for nonsynonymous mutations (*p* = 0.11) (Fig. 1C), suggesting that distinct forces influence nonsynonymous mutations across biological scales.

**Fig. 2:**
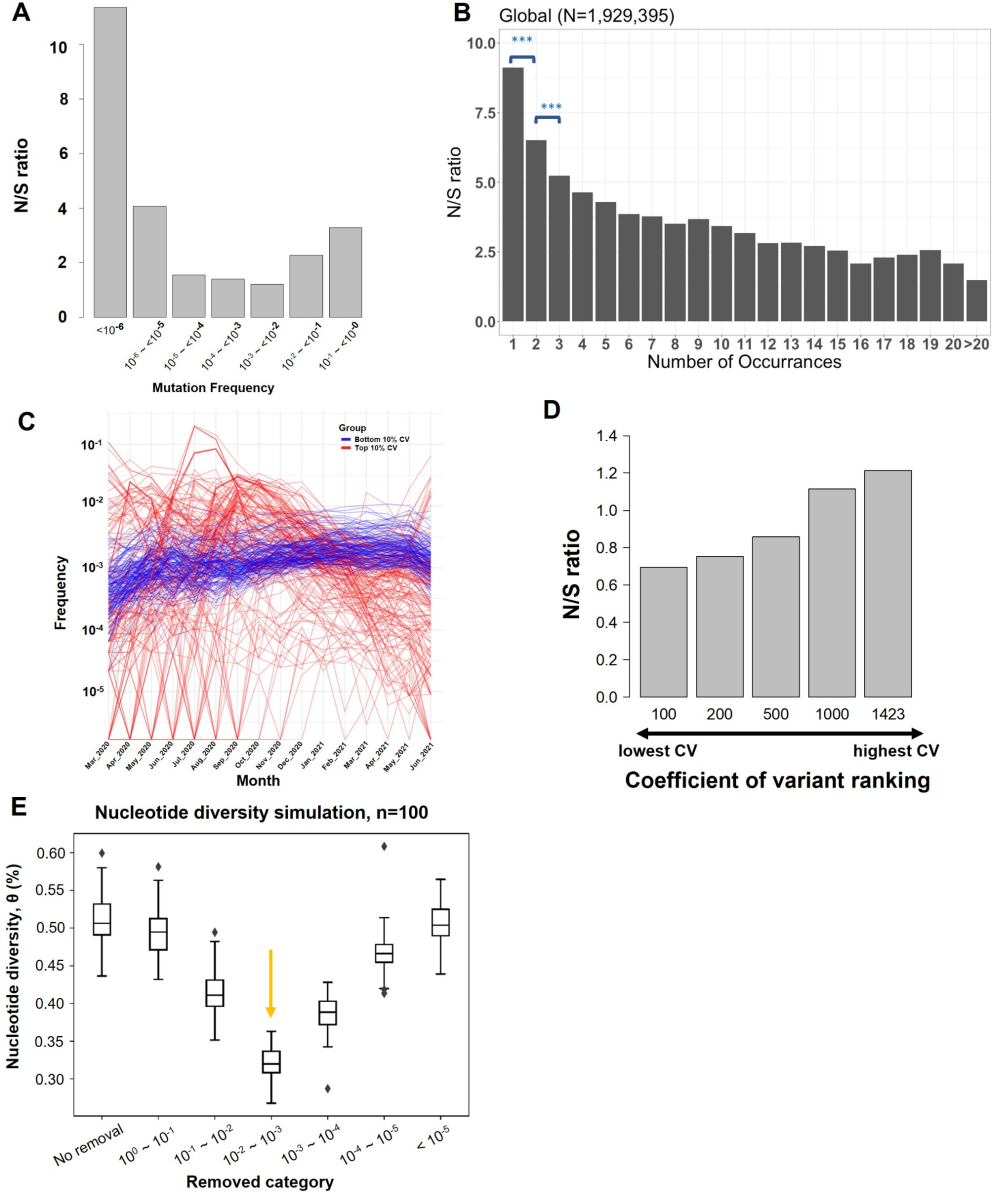
Nonsynonymous (N) and synonymous (S) mutations across frequencies. (**A**) N/S ratios of mutations at different frequencies. (**B**) N/S ratio of mutations that occurred 1–20 times in the population. The N/S ratio is highest with mutations that occurred once and rapidly decrease. Significant differences in mutation frequency are only observed between one-time and two-time occurrences, and between two-time and three-time occurrences. (*** *p* < 10^− 3^; Chi-square test). (**C**) Temporal dynamics of SARS-CoV-2 mutations with frequencies ranging from 10⁻³ to 10⁻², classified by their coefficient of variation (CV) over a 16-month period. Red lines represent the top 10% most variable mutations (highest CV), while blue lines indicate the bottom 10% least variable mutations (lowest CV). Each line shows the frequency trajectory of a single mutation. Although mutations in the top 10% display greater fluctuation, neither group exhibits a consistent upward trend, suggesting a lack of clonal expansion. (**D**) N/S ratios of mutations with frequencies ranging from 10⁻² to 10⁻³, which are significantly higher than those at four-fold degenerate sites, ranked by the coefficient of variation (CV) calculated from their monthly frequencies over a 16-month period. (**E**) Contribution of genetic diversity (θ) by mutations at different frequency. Watterson estimator of genetic diversity, θ (Watterson 1975; Y-axis), was estimated by randomly selecting 100 SARS-CoV-2 genomes from the dataset. Mutations at different frequency (X-axis) were individually removed to calculate the θ. The process was repeated 100 times and the mean and standard deviation of the θ were estimated. Although mutations within 10^− 3^– 10^− 2^ frequency bin only account for ~ 2% of total changes (indicated by an arrow), they represent ~ 37% of genetic diversity

To assess the strength of selection, we estimated the ratio of nonsynonymous (N) to synonymous (S) mutations. Across the entire genome, the N/S ratio was approximately 3.3 (22277.75 / 6735.25). For mutations with frequencies below 10^−^⁶, the N/S ratio was 11.37, which significantly dropped to 4.07 for mutations within the 10^−^⁶ to 10^−^⁵ frequency range bin (Fig. 2A; Table S3). To further explore the unexpectedly high N/S ratio in low-frequency mutations, we plotted the N/S ratios for mutations with frequency < 10^− 5^ (occurring < = 20 times in the population). The N/S ratio is highest for variants that occur once and rapidly decreases as the allele frequency goes up (Fig. 2B). Significant differences in mutation frequency are observed only between singletons and doubletons, and between doubletons and tripletons. A similar pattern is also observed when samples from distinct countries are plotted separately (Fig. S1). In all three countries (USA, UK, and Germany) where sample sizes exceed 100,000, the N/S ratio remains nearly flat for mutations that occur more than three times. High N/S ratios in singletons and doubletons suggest many of these nonsynonymous mutations are effectively “lethal” confined to one host and unable to spread (such as stop codon mutations; Supplement Text). It is worth noting that our analysis relies on sequences from public databases, which primarily capture high-frequency variations within hosts, as they are consensus sequences representing only the major nucleotides [25, 26]. This suggests that these mutations may be neutral or even confer benefits specifically to the hosts. This creates a dichotomy: what is advantageous inside a host becomes detrimental for between host transmission. As shown in Fig. 1C and supported by the high N/S ratios in singletons and doubletons (Fig. 2B), selection pressures within- and between-hosts appear to be decoupled or even in conflict.

### Pleiotropy maintains a large fraction of high frequency mutations

Mutations that are deleterious within hosts may not necessarily be so deadly during transmission and can sometimes rise to high frequencies within a population, potentially leading to pleiotropy. To pinpoint mutations with potential inverse pleiotropic effects, we began by identifying nonsynonymous variants with a significantly higher frequency than mutations at four-fold degenerate sites (> 10^− 3^) (Materials and Methods; black dashed line in Fig. 1A). These mutations may arise from mutation hotspots (mutation bias), be subject to positive selection, or be maintained by antagonistic pleiotropy. The latter is particularly relevant to viruses that cause acute infections. Figure 2A illustrates that N/S ratios increase for mutations with frequencies > 10^− 2^, suggesting that these nonsynonymous mutations would be advantageous and accumulate more rapidly than neutral (synonymous) counterparts. For variants with frequencies between 10^− 3^ and 10^− 2^, only synonymous mutation frequencies exhibit a strong positive correlation with intra-host variation (Fig. 1B), implying that while mutational bias may drive certain synonymous mutations to high frequencies, its impact on nonsynonymous mutations appears to be minimal.

An inverse pleiotropic effect arises when a mutation is beneficial in one context but detrimental in another. To investigate this, we ranked the 1,423 alleles with frequencies between 10^− 3^ and 10^− 2^ based on their coefficient of variation (CV) over a 16-month period, from lowest to highest. CV measures the dispersion of frequency distribution, smaller values indicate more consistent frequencies over time, implying that the mutation has not undergone significant clonal expansion or reduction (Fig. 2C). This is consistent with expectations under inverse pleiotropy. The N/S ratio for the top 100, 200, 500, 1000, and all 1423 sites, ranked from lowest to highest CV, is 0.69, 0.75, 0.86, 1.11, and 1.21, respectively (Fig. 2D). The high proportion of synonymous mutations with relatively stable frequencies during the pandemic indicates that most of these variants reflect the underlying mutational bias, as suggested in previous studies [27, 28]. However, the N/S ratio increases as frequency fluctuation goes up, indicating that nonsynonymous mutations in the top-ranking categories are being constantly created but never experience clonal expansion which in turn suggests that these variants are maintained by inverse or antagonistic pleiotropy.

### Pleiotropy is instrumental in maintaining viral genetic diversity and facilitating epistatic interactions

Figure 2 indicates that a significant number of nonsynonymous mutations with frequency > 10^− 3^ but < 10^− 2^ is probably maintained by pleiotropy. To illustrate its contribution to maintaining viral genetic diversity, we calculated Watterson’s estimator of genetic diversity, θ [18], by randomly selecting 100 SARS-CoV-2 genomes from the dataset and removing mutations from a set of frequency bins, one bin at a time (See Materials and Methods). Although the removal of variants did reduce genetic diversity as expected, it also highlighted the contribution of alleles across the frequency spectrum. For example, while mutations with frequency < 10^− 5^ constitute ~ 57% of total changes (Table S3), they only contribute to ~ 0.7% of genetic diversity (*p* = 0.06; t-Test; Fig. 2E). Conversely, although mutations with frequency ranging from 10^− 3^– 10^− 2^ account only for ~ 2% of total changes, they represent ~ 37% of genetic diversity.

Individually, these mutations are likely advantageous within hosts, yet exhibit marginal deleterious impacts during between-host transmission. Collectively, however, they might interact with each other to reduce the deleterious effect (antagonistic epistasis) [7], or even compensate and increase the overall fitness of the compound genotype (positive epistasis) [29, 30]. If this is true, it is reasonable to anticipate that combinations of these mutations (frequency ranging from 10^− 3^– 10^− 2^) can generate new adapted viral strains. Our inferences so far are based on SARS-CoV-2 genomes collected before July 5, 2021, prior to the emergence of the Omicron strain in late 2021. This allows us to test our conclusions by examining the fate in the later Omicron samples of variants that are in the 10^− 3^– 10^− 2^ frequency bin in our initial data set.

A significantly smaller than expected fraction of the 46 Omicron BA.2 specific amino acid changes is between 10^− 7^ and 10^− 5^ in frequency (*p* < 10^− 4^; Fisher exact test; Table S4). As discussed above, mutations with frequency < 10^− 5^ are likely to be deleterious, so a significantly lower number of nonsynonymous changes should be expected. In contrast, 12 out of 46 amino acid changes have frequencies > 10^− 2^ in our dataset (odds ratio = 254.5, *p* < 10^− 10^), consistent with the prediction that nonsynonymous mutations with frequency > 10^− 2^ may be advantageous. Importantly, 11 out of 46 nonsynonymous mutations fall with the 10^− 3^– 10^− 2^ frequency bin (odds ratio = 28.9, *p* < 10^− 11^), in line with our expectation that many of these variants are likely maintained by antagonistic pleiotropy and that they interact with each other to facilitate the emergence of a new successful viral strain.

### S-M1237I increases viral assembly and secretion but decreases infectivity

To test whether sub-high frequency mutations (> 10^− 3^ ~ < 10^− 2^) staying at relatively constant frequency over time are maintained by pleiotropy, we studied M1237I, which is one of the top-ranking mutations (Table S5, 25273T). Two mutations in the Spike protein, M1229I and M1237I, reside in or near the transmembrane domain (1214–1234 a.a.) of the protein’s cytoplasmic tail [31, 32]. This cytoplasmic tail, conserved between SARS-CoV and SARS-CoV-2 (Fig. S2), (identical in 38 out of 39 a.a.) might affect both the syncytium formation and viral entry, influencing viral infectivity [33, 34]. The exact role of M1237I is still unknown.

**Fig. 3:**
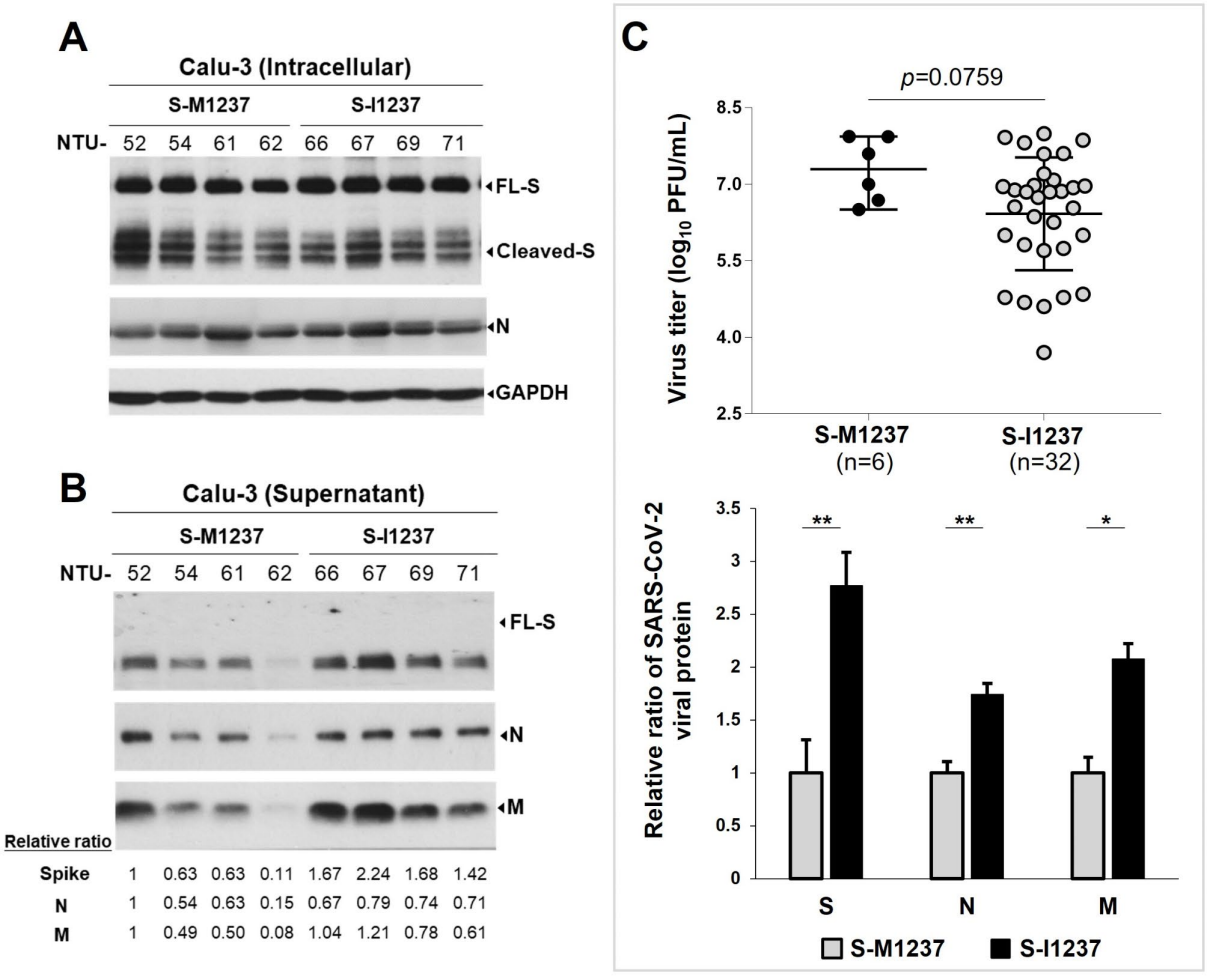
S-M1237I mutation in the SARS-CoV-2 Alpha strains associates with higher viral protein levels but lower plaque forming capability. (**A**)(**B**) Representative immunoblot results of viral protein expression in Calu-3 cells (**A**) and in supernatants (**B**) after 24 h post infection with SARS-CoV-2 isolates (S-M1237-Alpha and S-I1237-Alpha (T-III) variants, MOI = 0.1). The relative ratio of S, N, and M protein in the supernatant was normalized with NTU52 (set as 1). The relative ratios were listed below the immunoblot in left panel and graphically illustrated in right panel of (**B**). (**C**) Comparison of viral titers (plaque-forming units (PFU)/mL) in the supernatants of Calu-3 cells infected with SARS-CoV-2 isolates (S-M1237-Alpha and S-I1237-Alpha (T-III) variants, MOI = 0.1) at 24 h post-infection. Data are presented as the mean ± SD (*P* < 0.01^**^; *P* < 0.001^***^)

Our prior study identified a single SARS-CoV-2 lineage, T-III, responsible for Taiwan’s third outbreak (April 20-November 5, 2021) with four genetic markers, including spike M1237I [35]. To test the function of S-M1237I, viral titers (determined by the plaque forming assay) and viral protein amounts (determined by immunoblots of lysates from infected cells and from virions in the supernatant) for six SARS-CoV-2 S-M1237-Alpha and 32 S-I1237-Alpha strains (T-III) isolated at NTUH were compared using the Calu-3 cell culture-based virus infection system (Table S2). Representative immunoblots of intracellular lysates from virus-infected cells show similar levels of viral protein across virus groups (Fig. 3A, S3).

**Fig. 4:**
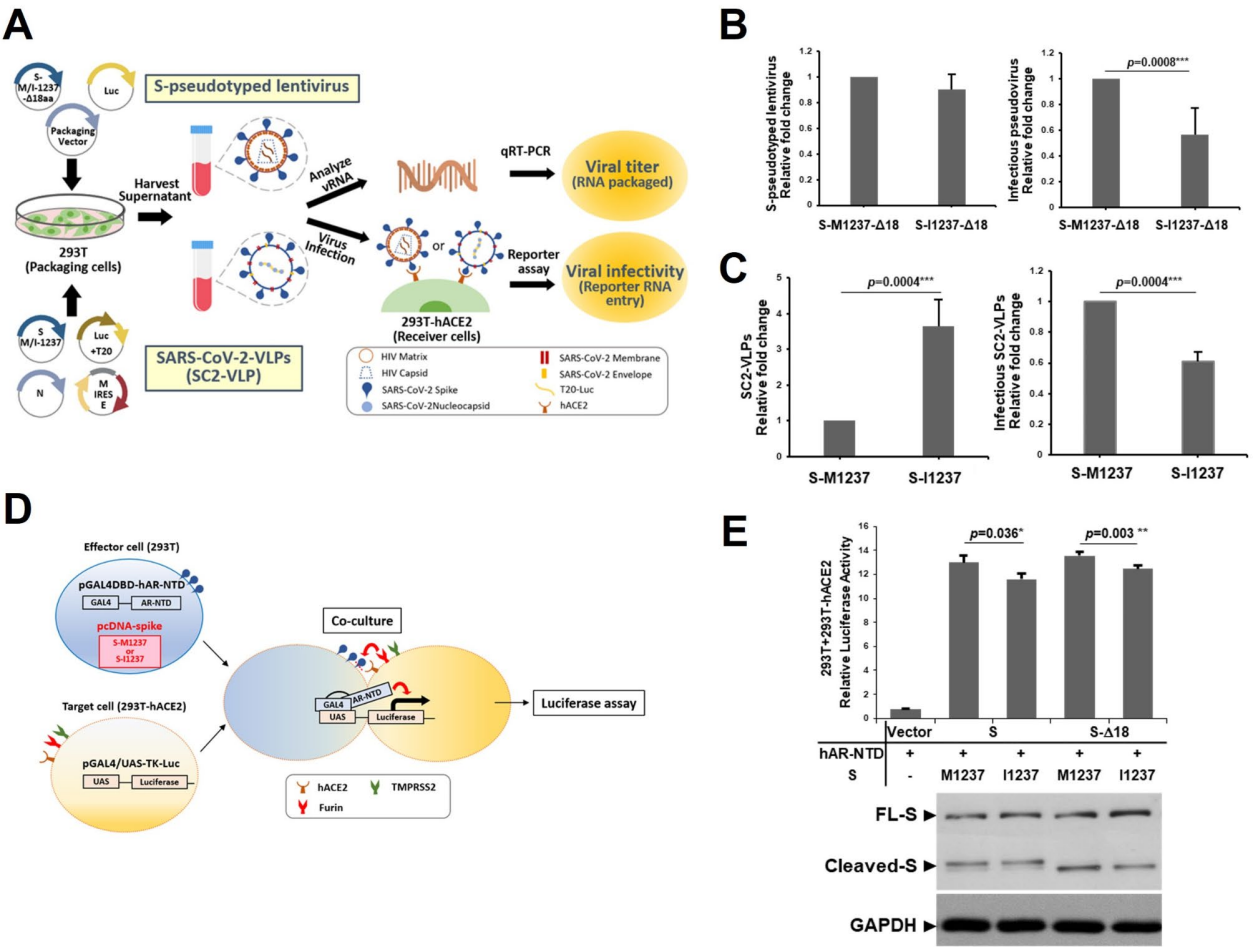
S-M1237I mutation increases viral assembly/secretion but decreases infectivity in vitro. (**A**) Schematic illustration of the protocols for generation of S-pseudotyped lentiviruses (upper panel) and SC2-VLPs (lower panel), and for subsequent viral titer and viral infectivity determination. (**B**) Analysis of the viral titer of the lentiviruses pseudotyped with S-B117-M1237- Δ18aa or S-B117-I1237- Δ18aa produced by 293T cells (left panel) and the viral infectivity (right panel, with S-B117-M1237- Δ18aa set as 1). The results were derived from six independent experiments and are shown as the mean ± SD (*P* < 0.05^*^). (**C**) Analysis of viral titers of the SC2-VLPs containing S-B117-M1237 or S-B117-I1237 produced by 293T cells (left panel) and viral infectivity (right panel, with S-B117-M1237 set as 1). The results were derived from three independent experiments and are shown as the mean ± SD (*P* < 0.05^*^). (**D**) Schematic illustration of the one-hybrid reporter assay for evaluating fusion activity induced by the SARS-CoV-2 S protein. Effector 293T cells were co-transfected with the expression plasmid for S-B117-M1237 or S-B117-I1237 and the pGAL4DBD-hAR-NTD plasmid. The target 293T-hACE2 cells were transfected with pGAL4/UAS-TK-Luc. At 24 h post transfection, the effector and target cells were co-cultured for 24 h and harvested to assay luciferase activity. (**E**) Representative results of the cell-cell fusion activity mediated by S-B117-M1237, S-B117-I1237, S-B117-M1237-Δ18aa or S-B117-I1237-Δ18aa, which were detected by the luciferase reporter assay of the lysates harvested from co-cultured cells (*P* < 0.05^*^) (upper panel). The results were derived from three independent experiments and are shown as the mean ± SD (*P* < 0.01^**^). The expression of the spike protein from 293T cells transfected with the indicated plasmids was analyzed by immunoblotting and GAPDH was included as a loading control (lower panel)

Compared to S-I1237-Alpha strains, S-M1237 isolates had less viral protein in supernatants but higher infectious titers (Fig. 3B, S4, 3C), suggesting more efficient assembly/release but lower infectivity of S-I1237 Alpha. To address this possibility and confirm the effect of the S-M1237I mutation itself, we tested two reporter viruses: SARS-CoV-2 spike pseudotyped lentivirus for infectivity (Fig. 4A, upper panel) and SC2-virus-like particles (VLPs) by Professors Doudna and Ott [23] to specifically evaluate S-M1237I’s impact on assembly/release and infection (Fig. 4A, lower panel). The pseudovirus experiment revealed that lentiviruses pseudotyped with S-B117-I1237 had significantly lower infectivity than S-B117-M1237 (Fig. 4B, right panel), despite equal virus release in the supernatant (Fig. 4B, left panel). In this assay, the C-terminal 18 amino acids of the ER retention signal of the spike protein were removed (Δ18aa) to optimize pseudotyped lentivirus production [21]. Since the only difference between the pseudotyped viruses is the M1237I amino acid change, the results directly implicate M1237I in decreasing viral infectivity.

We next generated SC2-VLPs with envelopes containing S-B117-M1237 or S-B117-I1237 and evaluated their assembly/release and infection. Expression plasmids for N, M, E, and either S-B117-M1237 or S-B117-I1237 were co-transfected with a packaging signal containing luciferase-encoding mRNA, Luc-T20, into the packaging 293T cells. qRT-PCR revealed more mRNA-containing SC2-VLPs released from S-B117-I1237 cells than S-B117-M1237 cells (Fig. 4C, left panel), suggesting S-M1237I may enhance SARS-CoV-2 assembly and release.

The mRNA-containing VLPs in the supernatant were then harvested for infection of the receiver ACE2-expressing 293T cells. We measured luciferase activity from the cells infected with the same amount of Luc-T20-containing SC2-VLPs (MOI = 0.05) to estimate infectivity rates. S-B117-I1237 containing SC2-VLPs show significantly less luciferase than S-B117-M1237 SC2-VLPs, indicating reduced infectivity (Fig. 4C, right panel). These results altogether suggest that S-M1237I increases assembly/release but reduces infectivity in T-III Alpha strain viruses.

To further investigate the underlying mechanism for this lower infectivity, we examined viral-cell membrane fusion mediated by spike-ACE2 interaction using the syncytium formation assay (details schematically illustrated in Fig. 4D). The S-B117-I1237 exhibited notably diminished syncytium formation compared to S-B117-M1237 in both full-length and Δ18aa spike proteins (Fig. 4E, S5). Given the importance of viral-cell membrane fusion for infectivity, our findings imply that S-M1237I reduces infectivity in T-III strains by affecting this fusion activity.

## Discussion

By analyzing 1,929,395 SARS-CoV-2 genomes, we found that while frequencies of synonymous mutations show a strong positive correlation with intra-host variation, this correlation is absent for nonsynonymous mutations (Fig. 1B and C). In addition, advantageous mutation within hosts might be deleterious during transmission between hosts, leading to a high N/S ratio among low frequency variants (Fig. 2). These findings hint at the possibility of pleiotropic effects arising from the dual selection pressures inherent in the viral life cycle, potentially reshaping our understanding of viral evolutionary dynamics. According to evolutionary theory, most mutations are deleterious and eliminated by negative selection. However, through pleiotropic effects, these mutations can persist in the population, as many are advantageous either within- or between-host, thereby sustaining a high level of genetic diversity. We found a set of variants (with frequencies 10^− 3^– < 10^− 2^) that, although not appearing to arise due to positive selection or elevated mutation rates (Fig. 2), are probably sustained by pleiotropy.

To test the above hypothesis, we studied the function of one such mutation, spike M1237I. We found that SARS-CoV-2 with the spike I1237 variant secretes more viral particles but has reduced infectivity compared to M1237. Demographically, M1237I is found in ~ 0.18% of all SARS-CoV-2 genomes analyzed (Table S6), indicating that it was repeatedly and frequently created. Within hosts, the spike I1237 variant of SARS-CoV-2 may experience a rapid increase in frequency due to its enhanced viral particle production. However, the reduced infectivity of this variant causes it to be outcompeted at the population level, resulting in its rapid elimination. If this explanation is correct, we would expect viruses carrying spike I1237 to continuously spread, providing that there is no other strain competing with it. Indeed, during the third outbreak of SARS-CoV-2 which resulted in 13,795 cases in Taiwan in 2021, all sequenced viruses carried spike I1237, demonstrating its capability for transmission. With no alternative strains present, the virus continuously spread within the community [35].

Interestingly, a recent study examining SARS-CoV-2 Omicron variants corroborates our findings on the pleiotropic effects of certain mutations. The mutation N679K in the spike protein, with a frequency of 1.17 × 10^− 3^ in our dataset, attenuates the virus in vitro and in vivo by increasing spike degradation. In addition, while N679K reduces viral pathogenesis, it facilitates replication in the upper airway, which may enhance transmissibility and contribute to Omicron emergence [36].

Our model can explain the formation of variants of concern (VOC) during SARS-CoV-2 evolution. Since the onset of COVID-19 pandemic, several waves of SARS-CoV-2 strains have been documented, spanning from the origin of D614G, Alpha, Delta, to Omicron. Other than D614G, the strain contains 17 (Alpha) to more than 50 (Omicron) mutations at the start of tracking. High diversity in the population, preserved by pleiotropy, can promote epistatic interactions among variants. These interactions may help mitigate the deleterious effects of the mutations or even compensate for each other’s deficits [7, 29, 30], thereby facilitating the emergence of novel, adaptive viral strains. Many of the VOC defining mutations have already been segregating in population prior to its emergence. By balancing its performance within- and between-host, a virus strain can evolve into a VOC through mechanisms such as hitchhiking or recombination. As shown in Table S4, while the overrepresentation of alleles in the > 10^− 2^ frequency bin among the Omicron strains may be due to advantageous effect of these changes, the significant enrichment the 10^− 3^– 10^− 2^ frequency variants is best explained by epistatic interaction among mutations maintained by pleiotropy.

Our model may also explain the discordant estimates of SARS-CoV-2 transmission bottleneck sizes in previous studies, ranging from 1 to 10 to 100–1,000 virions [16, 27, 37–39]. This discrepancy arises because the current methods for estimating transmission bottleneck size assume all mutations within hosts are neutral [8, 40]. Although selection coefficients and viral population sizes within hosts have been estimated in chronic HIV infection [41–45], the role of selection during inter-host transmission remains largely unexplored. Antagonistic pleiotropy implies that there are two layers of selection, i.e., within and between hosts, during virus evolution. The probability of a mutation being transmitted depends not only on the size of transmission bottleneck, as the neutral model predicts, but also on its effect at different stages, as demonstrated by our study of S-M1237I. Consequently, mutations with antagonistic pleiotropy would bias the estimation of bottleneck size. More importantly, although these changes only constitute ~ 2% of total mutations, they contribute ~ 37% of genetic diversity, significantly impacting population size estimates. As accurate quantification of transmission bottleneck size will help in predicting the rate of adaptation for a rapidly evolving pathogen such as SARS-CoV-2 [8, 9], it is essential to consider the effect of pleiotropy during viral transmission in the future.

## Conclusion

Our study suggests that viruses experience distinct selection pressures at the intra-host and inter-host levels, which in turn contribute to pleiotropy and genetic diversity. Our analysis indicates that mutations with frequencies between 10⁻³ and 10⁻², particularly those with low coefficient of variation (CV), are likely maintained by pleiotropy. To experimentally validate this hypothesis, we investigated the spike mutation M1237I, one of the lowest CV mutations, and found that while it enhances viral particle secretion, it reduces infectivity. This finding supports the idea that mutations beneficial within hosts may impose fitness costs during inter-host transmission. Moreover, mutations within the 10⁻³ to 10⁻² frequency range significantly contribute to maintaining viral genetic diversity, potentially facilitating the emergence of new variants through epistatic interactions. This highlights the need for further studies to assess the long-term evolutionary impact of these mutations on viral adaptation and transmission.

## Electronic supplementary material

Below is the link to the electronic supplementary material.

Supplementary Material 1

Supplementary Material 2

Supplementary Material 3

## Data Availability

All sequences used in this study can be downloaded from the GISAID database (https://www.gisaid.org/).
