# Supplementary material for: Tradeoffs between proliferation and transmission in virus evolution– insights from evolutionary and functional analyses of SARS-CoV-2
Source: Virol J. 2025 Apr 19;22:107. doi: 10.1186/s12985-025-02727-5 (PMC12008902; doi:10.1186/s12985-025-02727-5)
Supplement: Supplementary file 2 — Supplementary Material 2 [file 12985_2025_2727_MOESM2_ESM.pdf]

Tradeoffs between proliferation and transmission in virus evolution – insights from evolutionary and functional analyses of SARS-CoV-2

Jui-Hung Tai[1,2 †], Ding-Chin Lee[3 †], Hsin-Fu Lin[1], Tai-Ling Chao[4], Yongsen Ruan[5], Ya-Wen Cheng[3], Yu-Chi Chou[6], You-Yu Lin[1,2], Sui-Yuan Chang[4], Pei-Jer Chen[1,7,8,9], Shiou-Hwei Yeh[3*], Hurng-Yi Wang[1,2,10,11*]

Supplement Text

We also analyzed premature stop codon mutations within- and between-host. Because the potential advantageous nature of stop codon mutations in ORF8 (1), this ORF was excluded from the analysis. Within hosts, we identified 114 stop codon mutations, all with frequencies greater than 50% (Table A). Notably, only approximately 7% (8/114) of these mutations exhibited frequencies $>10^{-5}$ between hosts. In contrast, 35% of nonsynonymous mutations had frequencies $>10^{-5}$ between hosts (Table B; $p < 10^{-11}$). These findings suggest that while stop codon mutations may arise and even persist within a particular host, they are generally unsuccessful in spreading to other hosts, highlighting how genetic changes within a host often do not translate to inter-host transmission success.

Table A, Nonsense mutations across SARS-CoV-2 proteins

Protein[#]	AA_change	POS_ALT	intra-host count	inter-host frequency
E	K53*	26401_T	1	0
M	Y179*	27059_A	1	0.000000527
M	E11*	26553_T	1	0.000000517
M	K162*	27006_T	1	0
N	Q181*	28814_T	1	0.00000684
N	G5*	28286_T	1	0.0000021
N	K375*	29396_T	1	0.00000106
N	K248*	29015_T	1	0.00000105
N	W301*	29175_A	1	0.00000105

N	R10*	28301_T	1	0.000000534
N	K127*	28652_T	1	0.000000526
N	R36*	28379_T	1	0
orf1a	Q556*	1931_T	1	0.0000341
orf1a	L3430*	10554_A	1	0.0000236
orf1a	K47*	404_T	3	0.00000578
orf1a	K2478*	7697_T	1	0.00000317
orf1a	Q1332*	4259_T	1	0.00000315
orf1a	E995*	3248_T	1	0.00000262
orf1a	Q2348*	7307_T	2	0.00000211
orf1a	E36*	371_T	1	0.0000021
orf1a	Q2639*	8180_T	1	0.0000021
orf1a	E1766*	5561_T	1	0.00000158
orf1a	Q526*	1841_T	1	0.00000158
orf1a	E2585*	8018_T	1	0.00000158
orf1a	E1340*	4283_T	1	0.00000158
orf1a	Q3878*	11897_T	1	0.00000157
orf1a	R4150*	12713_T	1	0.00000105
orf1a	G697*	2354_T	1	0.00000105
orf1a	E3932*	12059_T	1	0.00000105
orf1a	E972*	3179_T	1	0.00000105
orf1a	E2*	269_T	1	0.000000536
orf1a	E87*	524_T	1	0.000000535
orf1a	Y4171*	12778_A	27	0.000000528
orf1a	E2970*	9173_T	1	0.000000526
orf1a	E1192*	3839_T	1	0.000000526
orf1a	E2088*	6527_T	1	0.000000524
orf1a	E1142*	3689_T	1	0.000000524
orf1a	E381*	1406_T	1	0.000000524
orf1a	Q3390*	10433_T	1	0.000000523
orf1a	S3874*	11886_G	2	0
orf1a	S2720*	8424_A	2	0
orf1a	S308*	1188_G	2	0
orf1a	W2837*	8776_A	2	0
orf1a	Y2331*	7258_G	1	0
orf1a	E102*	569_T	1	0
orf1a	K1902*	5969_T	1	0
orf1a	E1777*	5594_T	1	0
orf1a	K247*	1004_T	1	0
orf1a	S2220*	6924_A	1	0
orf1a	K1869*	5870_T	1	0
orf1a	S4119*	12621_A	1	0
orf1a	L1279*	4101_A	1	0
orf1a	E4143*	12692_T	1	0

orf1a	E888*	2927_T	1	0
orf1a	K1053*	3422_T	1	0
orf1a	Q2749*	8510_T	1	0
orf1a	K4348*	13307_T	1	0
orf1a	L2630*	8154_G	1	0
orf1b	E361*	14548_T	2	0.0000126
orf1b	Q1546*	18103_T	1	0.00000315
orf1b	C1976*	19395_A	1	0.00000166
orf1b	K1396*	17653_T	1	0.00000158
orf1b	Q2259*	20242_T	1	0.00000106
orf1b	E2661*	21448_T	1	0.00000106
orf1b	Q2252*	20221_T	1	0.00000105
orf1b	Q1166*	16963_T	1	0.00000105
orf1b	Q2555*	21130_T	1	0.00000105
orf1b	Q1441*	17788_T	1	0.000000534
orf1b	E2274*	20287_T	1	0.000000529
orf1b	W1683*	18516_A	1	0.000000526
orf1b	C942*	16293_A	1	0.000000526
orf1b	E269*	14272_T	1	0.000000525
orf1b	Q283*	14314_T	1	0.000000525
orf1b	G581*	15208_T	1	0.000000524
orf1b	K2232*	20161_T	3	0
orf1b	E787*	15826_T	2	0
orf1b	Y1043*	16596_A	1	0
orf1b	Q1589*	18232_T	1	0
orf1b	L2669*	21473_G	1	0
orf1b	W1872*	19083_A	1	0
orf1b	E1889*	19132_T	1	0
orf1b	E1124*	16837_T	1	0
orf1b	W2521*	21029_A	1	0
orf1b	K64*	13657_T	1	0
orf1b	R1171*	16978_T	1	0
orf3a	E242*	26116_T	2	0.0000673
orf3a	Q17*	25441_T	1	0.0000084
orf3a	Q116*	25738_T	1	0.00000158
orf3a	L203*	26000_A	2	0
orf6	E55*	27364_T	6	0.000200065
orf6	E13*	27238_T	1	0.0000132
orf7a	Q62*	27577_T	4	0.011998404
orf7a	Y18*	27447_A	2	0
orf7b	E33*	27852_T	1	0
orf8	Q27*	27972_T	9	0.464206312
orf8	E106*	28209_T	2	0.003954616
orf8	E59*	28068_T	3	0.00038542

orf8	G8*	27915_T	13	0.000293633
orf8	W45*	28027_A	1	0.000144814
orf8	Q29*	27978_T	1	0.0000644
orf8	L7*	27913_A	1	0.0000428
orf8	Y31*	27986_G	5	0.00000844
S	C1254*	25324_A	8	0.0000126
S	Q677*	23591_T	1	0.00000788
S	Q414*	22802_T	1	0.00000368
S	Q836*	24068_T	1	0.00000262
S	Q1180*	25100_T	1	0.00000262
S	Q409*	22787_T	1	0.00000158
S	S46*	21699_G	2	0.00000105
S	K1245*	25295_T	1	0.00000105
S	Y789*	23929_A	1	0.00000105
S	Q1002*	24566_T	1	0.00000105
S	K278*	22394_T	1	0.000000557
S	C301*	22465_A	1	0.000000553
S	K535*	23165_T	1	0.000000526
S	Y674*	23584_A	4	0
S	Q853*	24119_T	1	0
S	L1145*	24996_A	1	0
S	Y1206*	25180_G	1	0
S	Q1010*	24590_T	1	0
S	C1043*	24691_A	1	0
S	E1151*	25013_T	1	0

There are a total of 122 stop codon mutations. Eight of these mutations are found in ORF8. After excluding those in ORF8, the total number of stop codon mutations is 114.

Table B, Distribution of nonsense mutation within hosts and nonsynonymous nutation between hosts

	# of stop codon mutations within hosts	# of nonsynonymous changes between hosts	P-value*
Frequency < 10^{-5} between hosts	106	31187	
Frequency > 10^{-5} between hosts	8	16887	< 10^{-11}

* Fisher exact test

Reference

1. Wagner C, Kistler KE, Perchetti GA, Baker N, Frisbie LA, Torres LM, et al. Positive selection underlies repeated knockout of ORF8 in SARS-CoV-2 evolution. Nat Commun. 2024;15(1):3207.
