# Supplementary material for: Tradeoffs between proliferation and transmission in virus evolution– insights from evolutionary and functional analyses of SARS-CoV-2
Source: Virol J. 2025 Apr 19;22:107. doi: 10.1186/s12985-025-02727-5 (PMC12008902; doi:10.1186/s12985-025-02727-5)
Supplement: Supplementary file 3 — Supplementary Material 3 [file 12985_2025_2727_MOESM3_ESM.pdf]

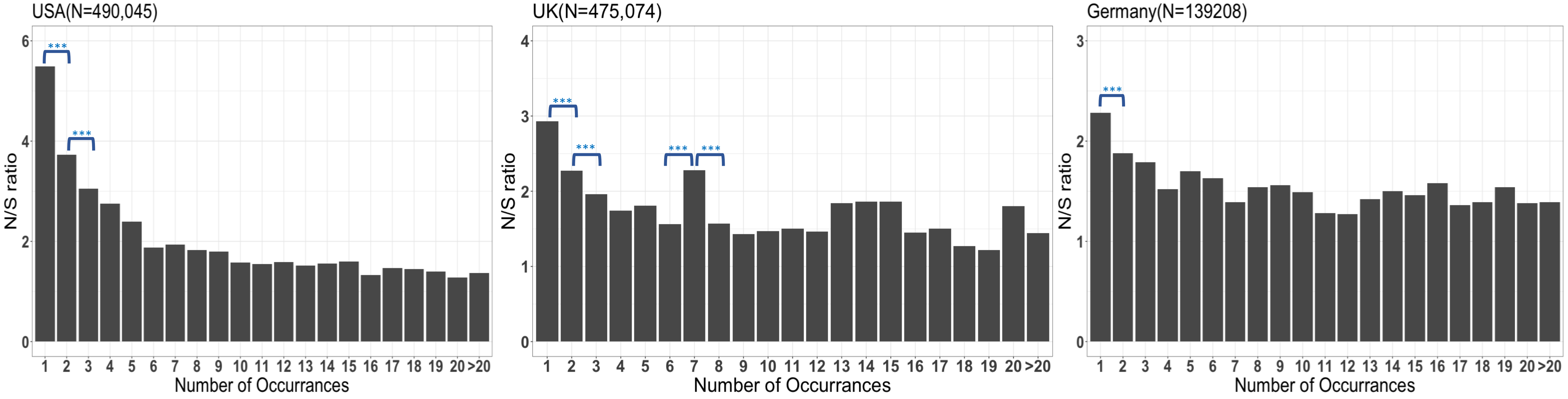

Fig. S1 N/S ratios of mutations that occurred 1 – 20 time in different countries. (*** $p < 10^{-3}$; Chi-square test)

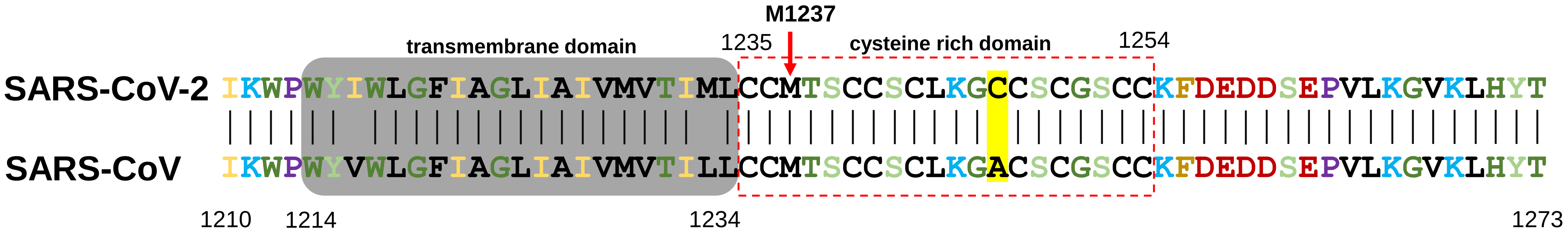

Fig. S2 The amino acid alignment of cytoplasmic tail of the SARS-CoV and SARS-CoV-2 Spike proteins.

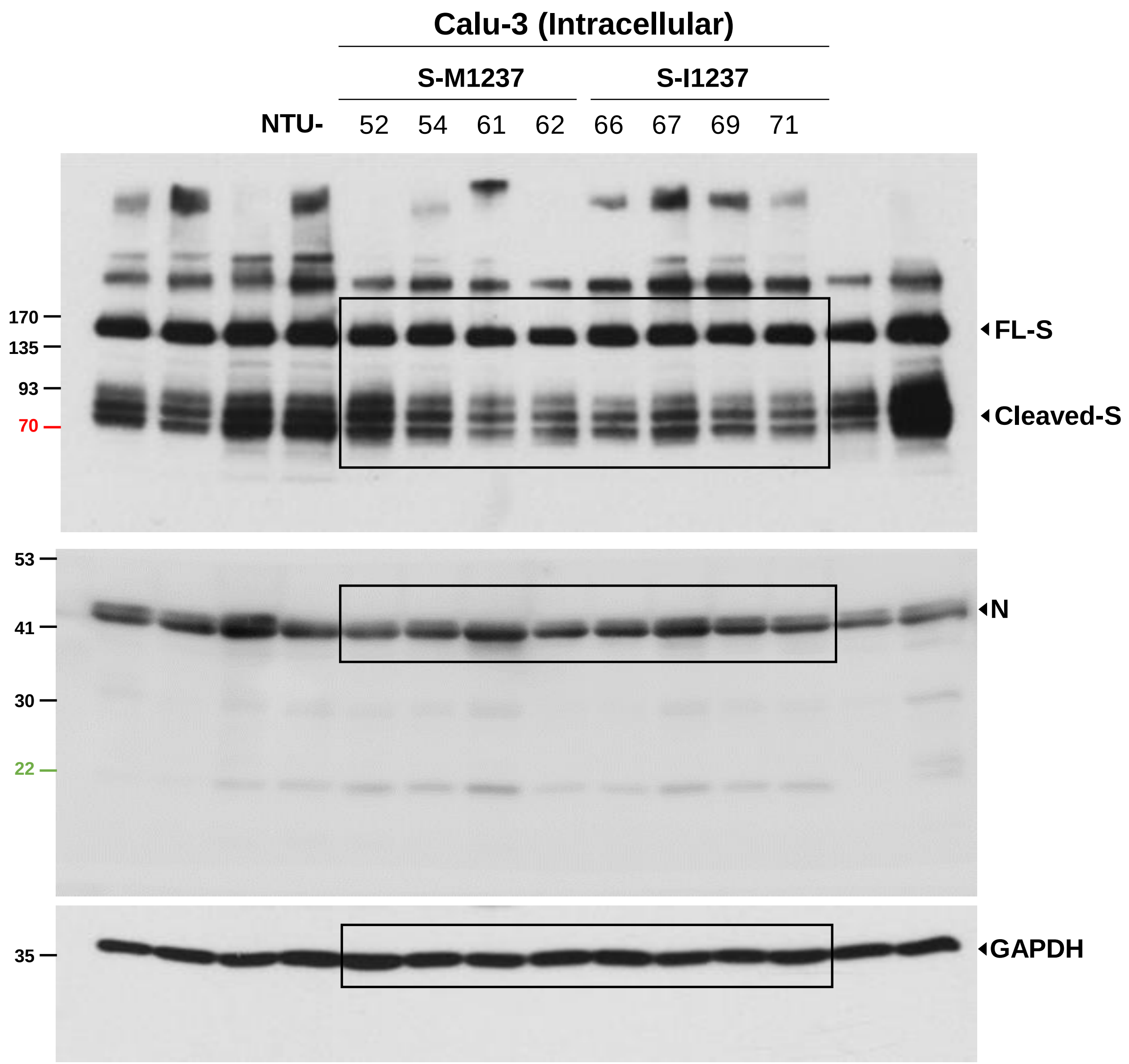

Fig. S3 Full length blot images that were cropped and presented in Figure 3A.

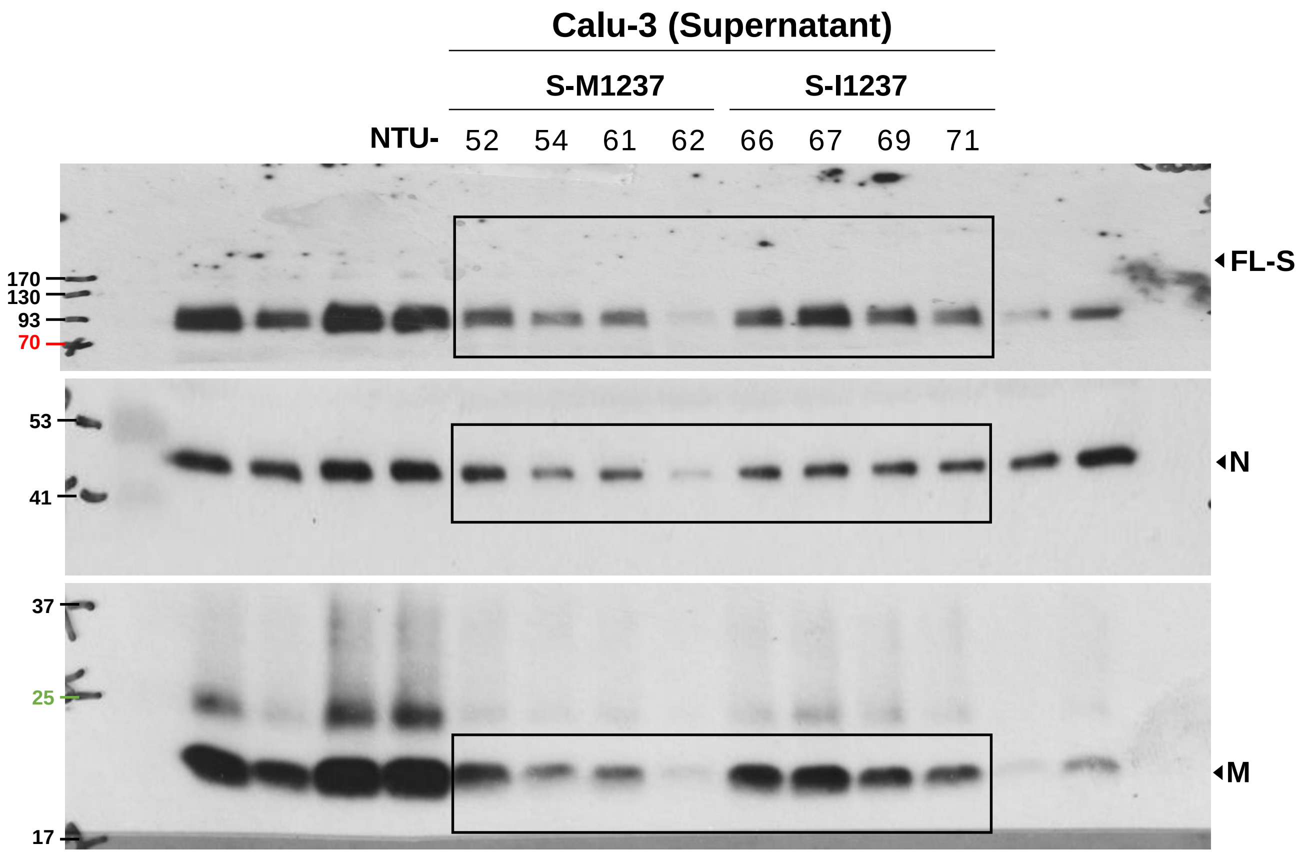

Fig. S4 Full length blot images that were cropped and presented in Figure 3B.

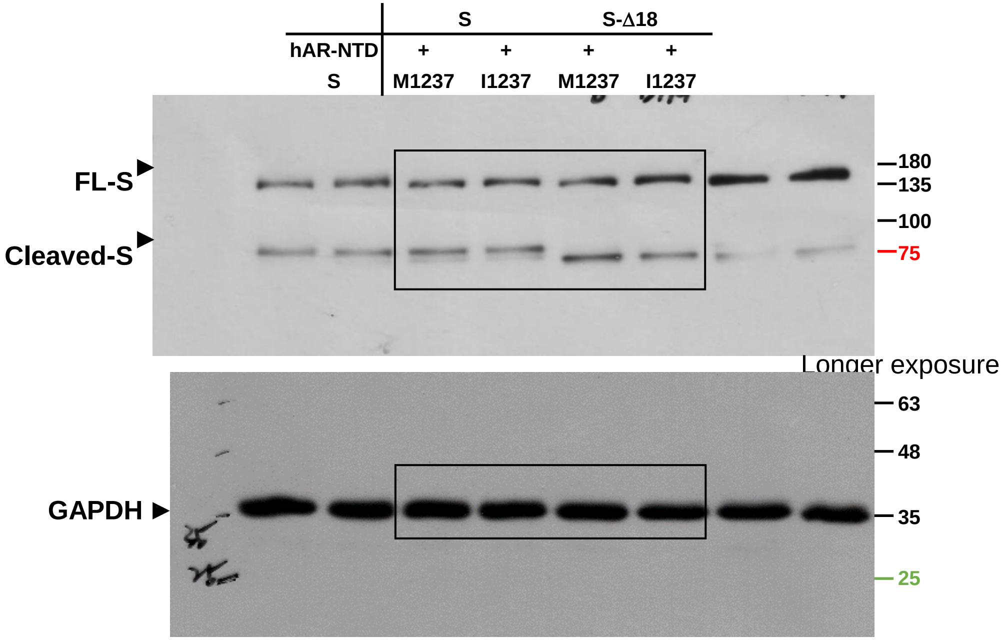

Fig. S5 Full length blot images that were cropped and presented in Figure 4E.
